# Preparation and Characterization of Chinese Leek Extract Incorporated Cellulose Composite Films

**DOI:** 10.3389/fbioe.2021.731749

**Published:** 2021-11-15

**Authors:** Qiying Zeng, Naiyu Xiao, Xueqin Zhang, Wenhan Luo, Gengshen Xiao, Wanjing Zhai, Le Zhong, Bifeng Lan

**Affiliations:** ^1^ College of Light Industry and Food Science, Zhongkai University of Agriculture and Engineering, Guangzhou, China; ^2^ Guangzhou Furui High Energy Technology Co., Ltd, Guangzhou, China

**Keywords:** microcrystalline cellulose, Chinese leek, packaging film, biocomposite, trifluoroacetic acid

## Abstract

This study aimed to prepare microcrystalline cellulose (MCC) films with good mechanical properties *via* plasticization using a Chinese leek (CL, *Allium tuberosum*) extract. The microstructure, crystal structure, mechanical properties, barrier ability, and thermal properties of the films were investigated. The chemical structure analysis of CL extract showed the existence of cellulose, lignin, and low-molecular-weight substances, such as polysaccharides, pectins, and waxes, which could act as plasticizers to enhance the properties of MCC:CL biocomposite films. The results of scanning electron microscopy and atomic force microscopy analyses indicated the good compatibility between MCC and CL extract. When the volume ratio of MCC:CL was 7:3, the MCC:CL biocomposite film exhibited the best comprehensive performance in terms of water vapor permeability (2.11 × 10^–10^ g/m·s·Pa), elongation at break (13.2 ± 1.8%), and tensile strength (24.7 ± 2.5 MPa). The results of a UV absorption analysis demonstrated that the addition of CL extract improved the UV-shielding performance of the films. Therefore, this work not only proposes a facile method to prepare MCC films with excellent mechanical properties *via* plasticization using CL extract but also broadens the potential applications of MCC films in the packaging area.

## Introduction

Currently, plastic has a wide range of applications in both manufacturing and daily life due to its excellent performance, ease of processing, and low cost. However, plastic particles, which may contain bisphenol A and o-phenyl plasticizers, can seriously affect the normal secretion and quality of human hormones (Yamamoto and Yasuhara, 1999). In Asian and European feces samples, 20 kinds of microplastic particles (50–500 μm) have been found in every 10 g of feces (Schwabl et al., 2019). Moreover, microplastics may translocate into gastrointestinal tissues and cause deleterious effects (Morgado et al., 2013; Deng et al., 2017). Thus, it is imperative to develop new and innovative materials, which should be renewable and environmentally biodegradable.

Cellulose is the most abundant renewable polymer found in nature. Because it is an unbranched crystalline polymer with a straight chain configuration, it can easily form strong fibers (Gumowska et al., 2019). However, the highly ordered intermolecular hydrogen bonding network of cellulose means that it exhibits stiff characteristics (in the absence of a plasticizer) (Zhang et al., 2020). One way to overcome this is by adding fillers such as a plasticizer. The addition of a plasticizer can reduce the brittleness and increase the flexibility of cellulose and can also help increase its water vapor permeability (WVP; Mahadevaiah et al., 2017). Earlier studies reported that plasticizers can be used in the manufacturing of cellulose-based packaging films. Common plasticizers found in cellulose films include epoxides, glycerol esters, phthalates, citrates, and so on (Rahman and Brazel, 2004; Schilling et al., 2010; Quintana et al., 2012; Müller et al., 2019). For example, a cellulose–polyvinyl alcohol biocomposite film plasticized with glycerol was prepared by Cazon et al. (2019). Their study showed that the use of a plasticizer in a composite film could increase the elongation at break but decrease the tensile strength. Moreover, the addition of glycerol enabled the composite film to exhibit a UV protective effect, which could prevent the oxidation deterioration of lipids in food when used in food packaging. Atta et al. (2021) developed edible food packaging films composed of yeast incorporated into bacterial cellulose (BC) and a mixture of carboxymethyl cellulose (CMC) and glycerol. Glycerol and yeast in the BC/CMC composite film acted as a plasticizer; this resulted in decreased tensile strength but an increased elongation at break. However, plasticizers (such as glycerol) in packaging materials might migrate out and be released into the food when applied to cellulose film packaging (Coltro and Machado, 2020). For example, Castle et al. (1988) found that 1.7–4.5 mg/kg of plasticizers were present in food as a result of migration. Tomita et al. (1977) indicated that the total levels of plasticizers ranged from 0.01 to 26 mg/kg in 55 samples of Japanese foods. Contact between food packaging and food may cause the plasticizers in the materials to transfer to the air. This phenomenon may contaminate the food, which eventually ends up inside the human body.

Trifluoroacetic acid (TFA) is a natural organic acid that is decomposed by microorganisms under aerobic and sulfur-free or anoxic conditions. TFA is a good solvent for biomacromolecules (e.g., proteins, polysaccharides, and cellulose) and can be easily recovered between −15 and 78°C through distillation or the use of a closed-loop system, thus making it environmentally friendly (Kim et al., 2000). The interaction between cellulose and TFA has been investigated by several previous studies. For example, Zhao et al. (2007) reported that TFA could penetrate the crystalline cellulose region and accelerate the decrystallization of cellulose, resulting in an amorphous structure of cellulose. Meanwhile, Guzman-Puyol et al. (2019) prepared nanocomposite cellulose materials by dissolving microcrystalline cellulose (MCC) and nanocellulose at room temperature in a mixture of TFA and trifluoroacetic anhydride, which could reduce the crystallite size of cellulose and increase the amorphous phase content of cellulose nanocomposite. Hasegawa et al. (1992) dissolved prepared cellulose and chitosan films in TFA, which turned partly trifluoroacetylated cellulose in a solution state into partly acetylated cellulose in a solid state during evaporation of the solvents in air. Therefore, TFA is an excellent solvent for solubilizing cellulose with other biomacromolecules.

On the other hand, Bayer et al. (2014) reported that TFA could be used as a solvent to form bioplastic films with tunable mechanical properties from vegetable waste. Thus, inspired by previous research, this study aims to use Chinese leek (CL) extract as a plasticizer for cellulose to develop the properties of MCC:CL biocomposite films using TFA as a solvent. Moreover, this study attempts to evaluate the influence of CL addition on the morpho-structural [scanning electron microscopy (SEM), atomic force microscopy (AFM), Fourier-transform infrared (FT-IR), and X-ray diffraction (XRD)] properties, WVP, mechanical properties, and thermal stability of the developed films. The UV absorption properties of the MCC:CL biocomposite films are also evaluated. Biocomposite films derived from vegetable extracts and cellulose can be a part of the new bioplastic generation that significantly reduces the harm caused by plastics to the environment and human health and promotes the utilization ratio of resources.

## Methods

### Materials

TFA and MCC were obtained from Macklin Biochemical Technology Co., Ltd. (Shanghai, China). CL was simply taken from the local vegetables market.

### Preparation of Film

The renewable film was prepared using a casting/evaporation method. Cleaned CL was pulverized and then dried in an oven at 60°C for 6 h. CL solution (3 wt.%) was then prepared by dissolving the resulting powder in TFA for 3 days. The mixture was then centrifuged (800°r/min, 5 min), after which the residuals were removed through filtering (400 meshes). The film was prepared by casting/evaporating 40 ml CL solution on round polyfluortetraethylene plates with a diameter of 12 cm and then dried in a fume cupboard. After that, the film was stored at a relative humidity of 60% for 2 days to remove any residual TFA. The MCC film was produced using the same method as described above.

### Preparation of MCC:CL Biocomposite Films

**SCHEME 1:**
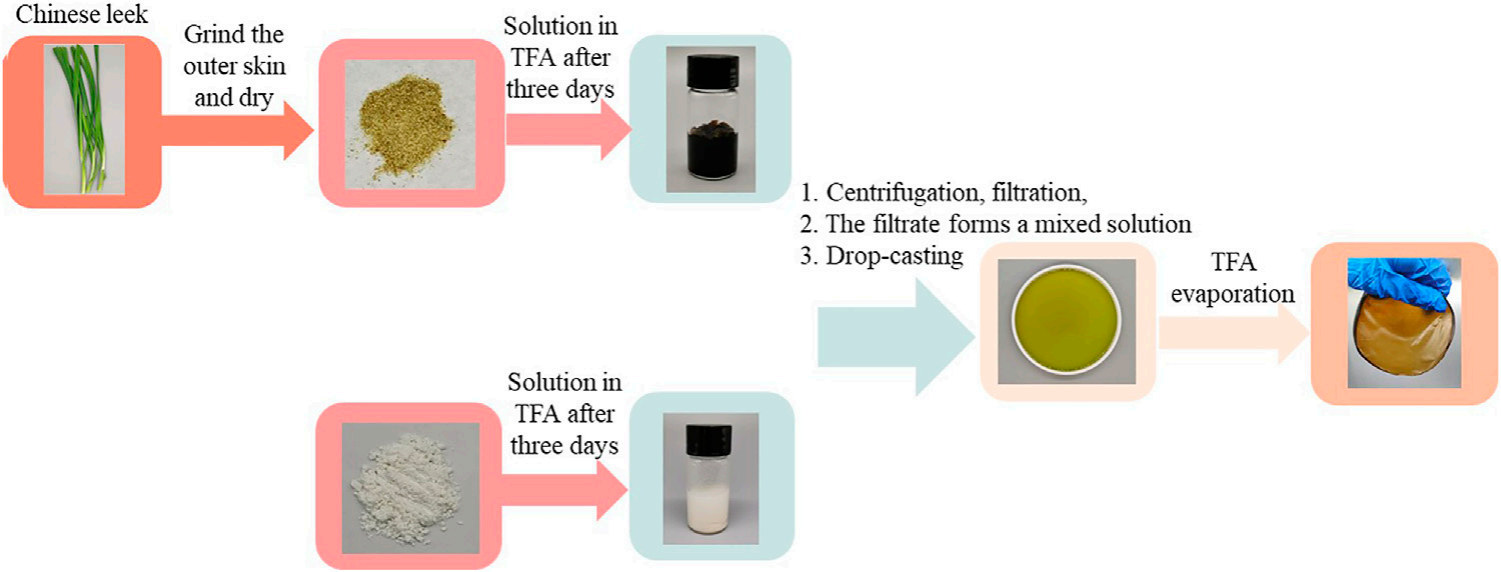
Preparation of MCC:CL biocomposite film

The preparation of MCC: CL biocomposite film is shown in Scheme 1. CL solution (3 wt.%) and MCC solution (3 wt.%) were prepared by dissolving in TFA for 3 days, respectively. The resulting solutions were separated by centrifuging (800 r/min, 5 min), after which the residues were removed through filtering (400 meshes). The filtered CL solution was added to the MCC solution with the volume ratios ranging from 10, 30, to 60% (v/v). These films were prepared by casting/evaporating 40 ml composite solution on round polyfluortetraethylene plates with a diameter of 12 cm, and then dried in a fume cupboard. After that, the films were stored at a relative humidity of 60% for 2 days to remove any residual TFA.

### Characterization

Nuclear magnetic resonance (NMR) was performed with a Bruker Avance Ⅲ 600-MHz spectrometer (Germany) equipped with a 5-mm QCI cryoprobe with a z-shielded pulsed-field gradient coil using 5-mm tubes filled with 500 µl of the sample solution. 90–100 mg of the samples was dissolved in 1 ml DMSO-d6.

Gel permeation chromatography (GPC, PL-GPC 50, Agilent) with a column (PLgel Olexis) was applied to measure the molecular weight (M_W_) of the CL extract. DMSO (HPLC grade) was used as the eluent. The GPC system was calibrated by a series of polysaccharide standards with polydispersity of 1.4.

FT-IR spectra were characterized using a Perkin–Elmer Spectrum 100 spectrometer. Sixty-four scans were collected over a wavelength range of 4,000–400 cm^−1^ at a resolution of 4 cm^−1^ in the attenuated total reflection mode.

A SEM analysis was obtained on JSM-6330F (Japan). The micelles were dropped on a metal stub using carbon tape and coated with gold. The wet films were frozen in liquid nitrogen, immediately snapped, and then freeze-dried to observe their cross-sectional morphology.

AFM images were acquired with a MultiMode 8 scanning probe microscope, and acquired images in the air using a vibration isolator and a soundproof cover. A single-beam silicon cantilever tip was used for data acquisition, with a nominal radius of less than 10 nm, an elastic force constant of 42 N/m, and high sensitivity. The resonance frequency was defined around 290 kHz. Depending on the sample roughness, the scan rate was between 0.5 and 1.0 Hz.

The XRD patterns were obtained using a Rigaku D-MAX 2200 VPC X-ray diffractometer using high-intensity monochromatic nickel-filtered Cu Kα radiation (λ = 0.15418 nm) generated at 40 kV and 40 mA. The diffraction angles (2θ) were in the range of 5°–40°. The step size was 0.04°.

The mechanical properties of the films were determined using an Instron GBU-1 Universal Testing Machine with a load cell of 100 N, conforming to the “ASTM D882-12” standard. The films were cut into rectangular strips measuring 15 × 10 mm, such that the test could be repeated three times for each sample. The grip length was set to 30 mm. The samples were used to produce a stress–strain curve at a strain rate of 250 mm/min and thus derive the tensile strength, Young’s modulus, and elongation at break.

The thickness was measured at five random locations on each sample using a digital micrometer Mitutoyo (Japan).

The UV absorption properties of the film was determined by absorption spectroscopy using a UV–visible spectrum (China). The transmittance date was used to calculate the ultraviolet protection factor (UPF) using Eq. 1

$$\text{ UV protection factor }\left(\text{UPF}\right)=\;\frac{\int_{290}^{400}E\left(\lambda \right)\;S\left(\lambda \right)\;d\lambda }{\int_{290}^{400}E\left(\lambda \right)\;S\left(\lambda \right)\;T\left(\lambda \right)\;d\lambda }.$$ (1)

The UPF value is used to estimate how much the material reduces UV exposure. Here, E(λ) is the relative erythema action spectrum, S(λ) is the spectral irradiance (W⋅m^−2^W⋅nm^−1^), T(λ) is the average spectral transmittance of fabric, dλ is the bandwidth, and λ is the wavelength.

The percentage blocking for UV-A (320–400 nm) was calculated by Eq. 2.

$$\text{UV}-\text{A}\;\text{blocking}\left(\%\right)=\text{100}-\;\frac{1}{\;m}\;\overset{400}{\underset{\lambda =320}{\sum }}T\left(\lambda \right),$$ (2)

where, m is the times measured between 320 and 400 nm.

The percentage blocking for UV-B (290–320 nm) was calculated by Eq. 3.

$$\text{UV}-\text{B}\;\text{blocking}\left(\%\right)=\text{100}-\frac{1}{\;k}\overset{320}{\underset{\lambda =290}{\sum }}T\left(\lambda \right),$$ (3)

where, k is the times measured between 290 and 320 nm.

The WVP of the films was determined according to Zhang et al. (2020). The films were sealed on a test vessel with a diameter of 28.0 mm and an exposed area of 615.44 mm^2^ was used for the study. It was filled with 40 g of dried silica gel and sealed with the films. Then the bottles were placed in a desiccator containing water at 20°C. The bottles were weighted periodically at intervals of 24 h for 7 days. WVP was calculated according to Eq. 4

$$\text{WVP}=\frac{w\times L}{t\times A\times \Delta P},$$ (4)

where, w (g) is the weight gained for 7 days, t (s) is the elapsed time, A (m^2^) is the film permeation area, L (m) is the film thickness, and ∆P is 2,339 Pa at 20°C.

The thermal stability of the films was tested using a Mettler Toledo TGA-2, by applying a standard thermogravimetric analysis (TGA) method. The measurements were performed on 10–20 mg of the samples in an aluminum pan under an inert nitrogen atmosphere with a heating rate of 10°C/min over a temperature range of 30–600°C. The weight loss (TG curve) and its first derivative (DTG curve) were recorded as a function of time and temperature. The weight loss (TG curve) and its first derivative (DTG curve) were recorded simultaneously as a function of time/temperature.

### Statistical Analysis

All the experiments were conducted at least three times and expressed as mean ± SD. A significance analysis was performed by the least significant difference (*p* < 0.05), and Origin 8.5^1^ was used for graph drawing.

## Results and Discussion

### Chemical Structure of CL Extract

**FIGURE 1:**
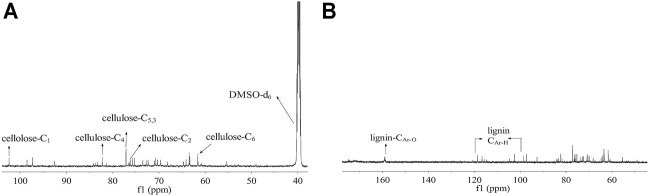
^13^C-NMR spectra of CL extract in DMSO-d_6_; ^13^C-NMR spectra of **(A)** cellulose and **(B)** lignin in the CL extract.

In order to analyze the ingredient in CL extracted with TFA, the chemical structure of the CL extract was elucidated by NMR. The ^13^C-NMR spectra of the CL extract (Figure 1A) shows peaks at 61.6 (C_6_), 76.2 (C_2_), 77.1 (C_5,3_), 82.2 (C_4_), and 102.4 (C_1_) ppm, which are attributed to the typical carbons of the anhydroglucose in cellulose (Hasegawa et al., 1992). Moreover, as shown in Figure 1B, the signals located at 158.8 (C_Ar-O_) and 125-103 (C_Ar-H_) ppm were assigned to the p-hydroxyphenyl moieties (C_Ar-O_) and aromatic methine carbons (C_Ar-H_) in lignin, respectively (Capanema et al., 2004). These results indicate the existence of cellulose and lignin in the CL extract.

**FIGURE 2:**
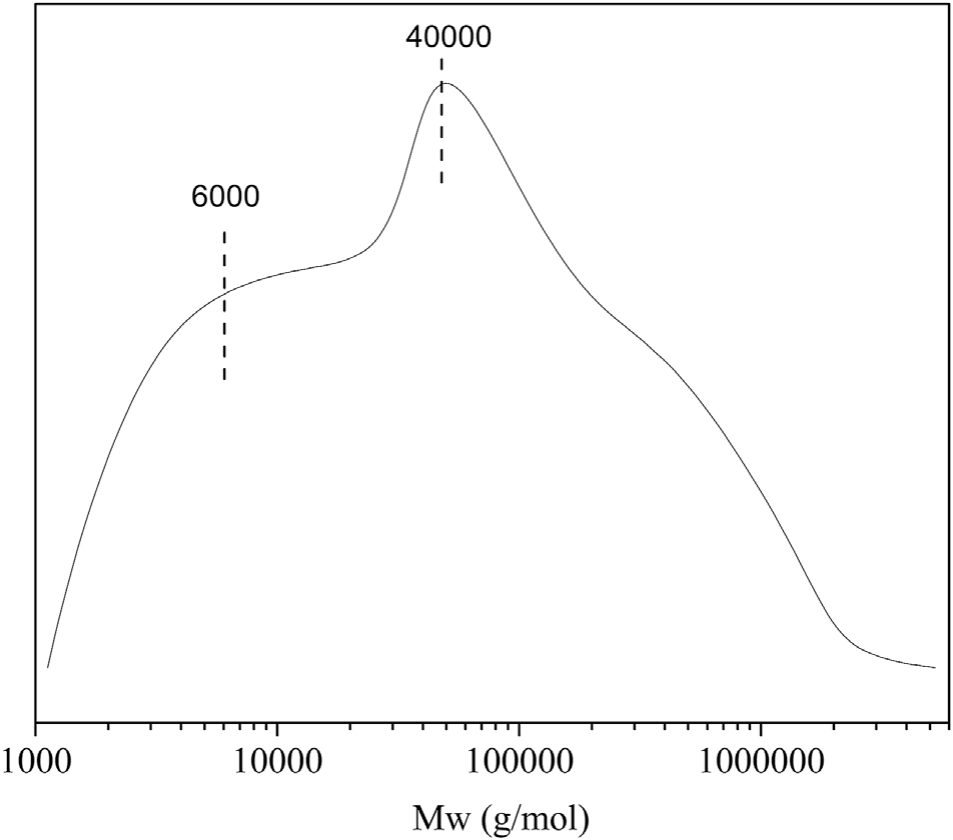
The molecular weight distributions of CL extract.

Molecular weight distribution of the CL extract was analyzed by GPC (Figure 2). The result shows that the molecular weight distribution of the CL extract is relatively wide, indicating that complex substances may be extracted by directly immersing CL in TFA. As shown in Figure 2, the CL extract shows two main peaks, with the average molecular weights of 40,000 and 6,000 g·mol^−1^, respectively. The peak at 40,000 g·mol^−1^ is probably attributed to amorphous cellulose, hemicellulose, and lignin by direct extraction with TFA (Unterweger et al., 2014). The peak at 6,000 g·mol^−1^ is probably assigned to the low-molecular-weight substances such as polysaccharides, pectins, and waxes (Timpa, 1991). In terms of these, CL extract plays an important plasticization role in the biocomposite film due to the extracted low-molecular-weight components, showing good plasticization potential as compared with plasticizers such as glycerin, ethylene glycol, wax, and xylitol.

### Microstructure Analysis of MCC:CL Biocomposite Films

**FIGURE 3:**
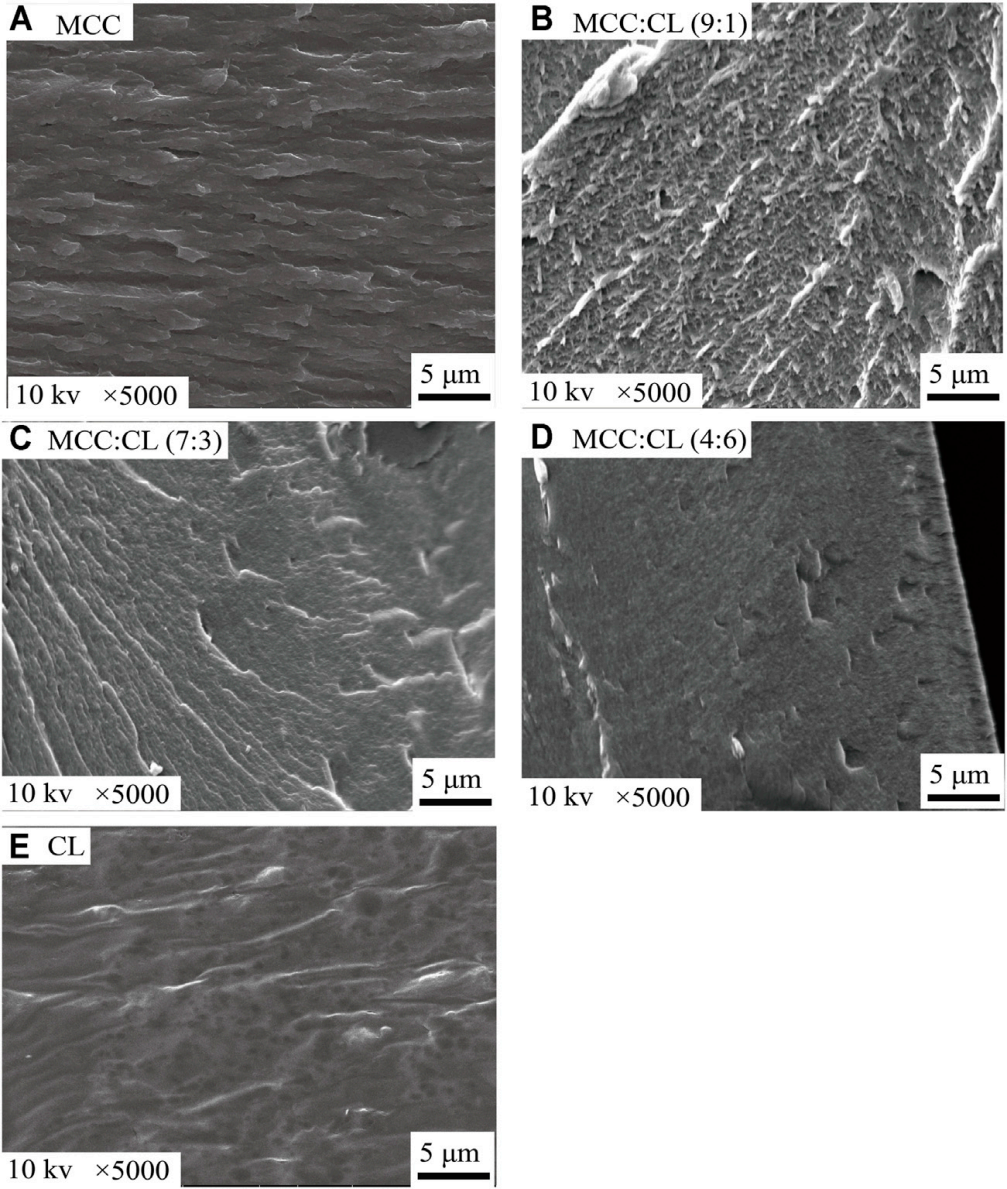
SEM images of fractured cross sections of the MCC film **(A)**, MCC:CL (9:1) film **(B)**, MCC:CL (7:3) film **(C)**, MCC:CL (4:6) film **(D)**, and CL extract **(E)**.

The SEM (as shown in Figure 3) images of the cross section of the MCC:CL biocomposite films were observed to study the interfacial morphology between MCC and CL extract since the mechanical properties depend on interfacial morphology (Kemala et al., 2012). The MCC film (Figure 3A) indicated its coarse-layered structure. The SEM image (Figure 3E) of the CL extract revealed the compact and smooth structure, probably because the aggregation of cellulose, lignin, grease, polysaccharides, pectin, and waxes from CL may generatein the TFA solution during the film formation (Morgado et al., 2013; Basiak et al., 2016; Gao et al., 2018). With the increase of the CL extract in the MCC:CL biocomposite films (Figures 3B–D), the cross section of the biocomposite film exhibited a smooth and more homogeneous fibrous surface instead of obvious cracks or breaks, indicating that no phase separation was observed between the CL extract and cellulose (Teaca et al., 2013).

**FIGURE 4:**
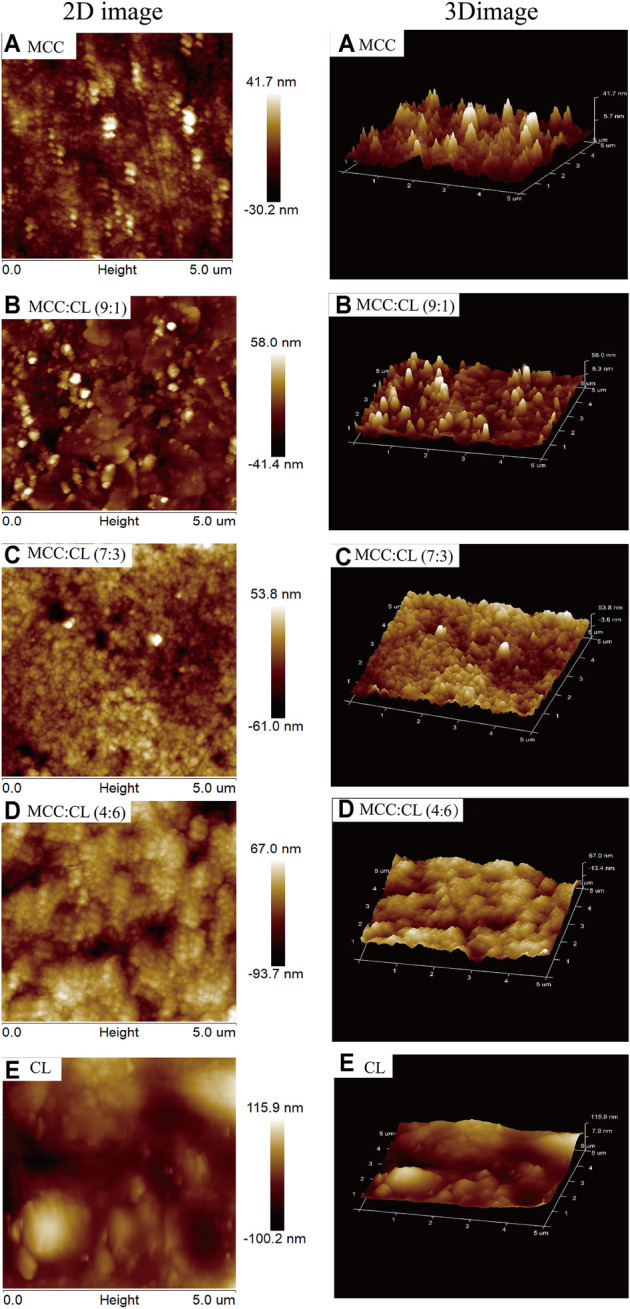
Surface morphologies of AFM of the MCC film **(A)**, MCC:CL (9:1) film **(B)**, MCC:CL (7:3) film **(C)**, MCC:CL (4:6) film **(D)**, and CL extract **(E)**.

The surface morphology and root mean squared (RMS) roughness (calculated over an area of 25 μm^2^) of the films were investigated using AFM. The AFM image of the MCC film showed a surface that was relatively less rough. The RMS of the MCC film as the control was recorded to be approximately 71.9 nm. However, it could clearly be observed that the incorporation of the CL extract into the MCC matrix significantly changed the surface morphology of the pure MCC film. The RMS values of the MCC:CL (9:1), MCC:CL (7:3), and MCC:CL (4:6) films were confirmed as 99.4, 114.8, and 160.7 nm, respectively, indicating that the incorporation of the CL extract increases the surface roughness. Clusters between MCC and CL extract were observed in the MCC:CL biocomposite films (Figures 4B–D), which became increasingly evident with increased CL extract content. The cluster features are interpreted to be the result of MCC aggregation or CL extract–coated MCC. Similar results have been reported in an earlier study by Haafiz et al. (2013).

### Crystal Structure Analysis of MCC:CL Biocomposite Films

**FIGURE 5:**
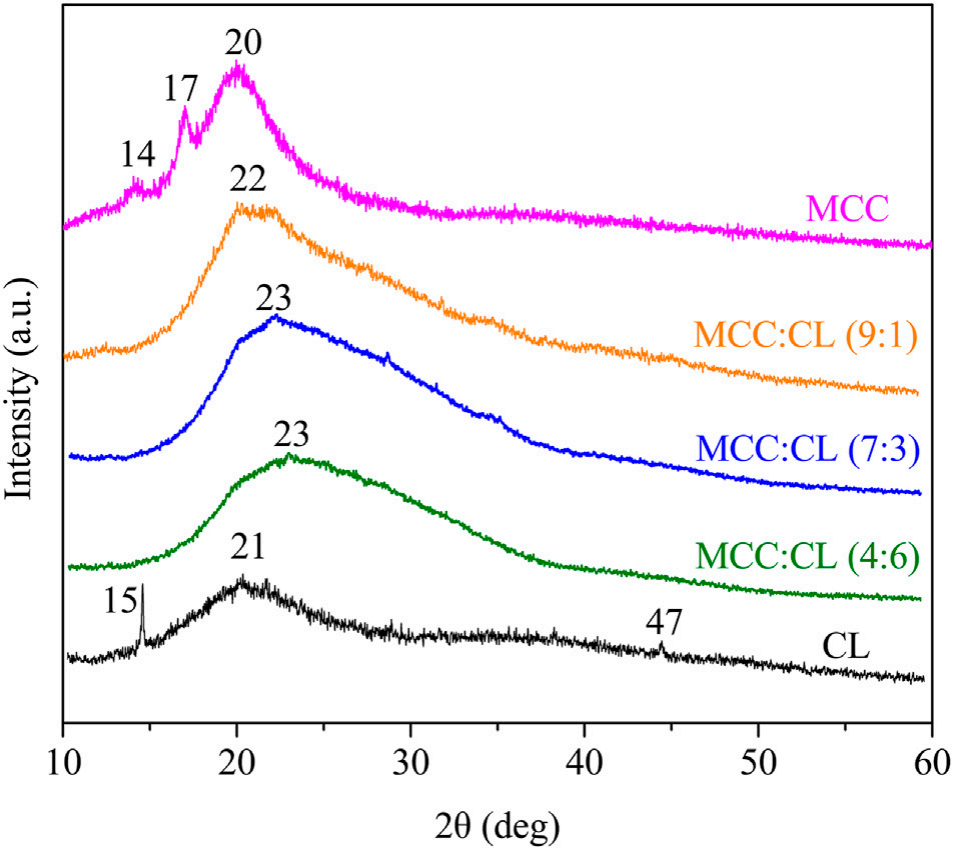
X-ray diffraction patterns of the MCC film, CL extract, and MCC:CL biocomposite films.

Figure 5 shows the XRD patterns of the MCC film, CL extract, and MCC:CL biocomposite films to determine the crystal structure of the film and research the effect of the CL extract on the MCC:CL biocomposite films on the crystal structure of the MCC film.

The XRD peak of the MCC film appearing at 2θ = 20° was attributed to a cellulose structure. Furthermore, the peaks at approximately 2θ = 14° and 17° were attributed to crystalline cellulose Ⅰ (Sèbe et al., 2012). The amorphous nature of the CL extract was confirmed by the existence of a very broad peak at 2θ = 21°. The XRD observations of the CL film revealed the diffraction peaks at 2θ = 15°, 21°, and 47°, which can be attributed to the ionic crystals in the CL extract (Kirinovic et al., 2017). Some ionic crystals present in the CL were introduced into the CL extract during the preparation of the CL solution. This result is similar to the report obtained by Bayer et al. (2014). By comparing the XRD pattern of the MCC:CL biocomposite films with a pure MCC film, the intensity of 2θ = 20° decreased and shifted to a higher region by increasing the CL volume ratio. This phenomenon occurs because of a higher content of CL in the material, which makes the MCC:CL biocomposite film more amorphous and hence modifies the microstructure of the MCC:CL film. This is in agreement with a previous study (Su et al., 2010).

### Mechanical Properties of MCC:CL Biocomposite Films

**FIGURE 6:**
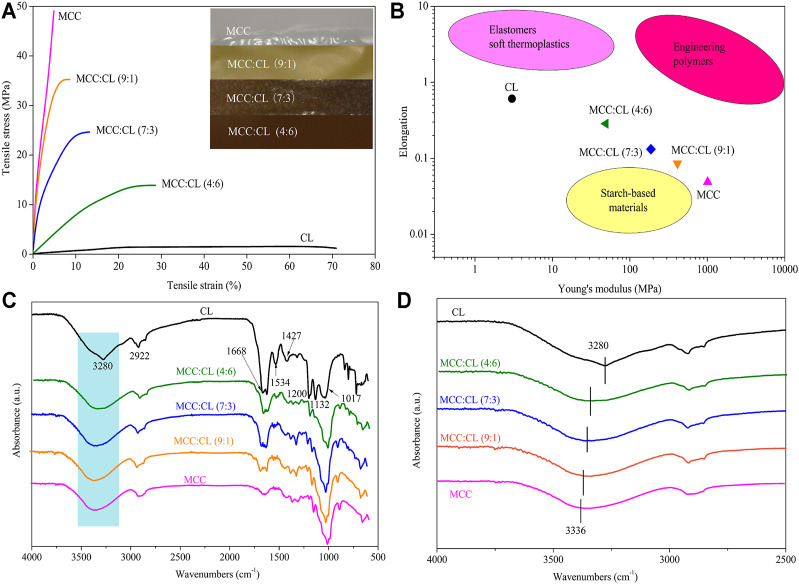
Typical stress–strain curves for MCC:CL blends **(A)**; the photograph in figure **(A)** shows the MCC film and CL extract with different ratios; elongation and Young’s modulus data of MCC:CL blends **(B)**, compared with the ranges of performance of elastomers/soft thermoplastics, starch-based materials, and engineering polymers; FT-IR spectra **(C)** of the MCC film, CL extract, and MCC:CL blends; FT-IR spectra **(D)** of MCC:CL blends in the hydroxyl group stretching region.

Mechanical properties, including the tensile strength and elongation at break of the MCC:CL biocomposite film, are determined relative to MCC and CL extract (reference), and the results are shown in Table 1 and Figure 6A.

**TABLE 1:** Tensile properties of the MCC film, CL extract, and MCC:CL biocomposite films.

Sample	Thickness (μm)	Tensile strength (MPa)	Elongation at break (%)	Young's modulus (MPa)
MCC	91.0 ± 1.0	49.1 ± 3.5	4.9 ± 1.2	1,010.0 ± 52.0
MCC:CL (9:1)	86.0 ± 2.0	35.3 ± 2.7	8.6 ± 1.2	411.0 ± 30.0
MCC:CL (7:3)	62.0 ± 3.0	24.7 ± 2.5	13.2 ± 1.8	187.0 ± 21.0
MCC:CL (4:6)	50.0 ± 4.0	13.9 ± 2.2	28.6 ± 2.7	49.0 ± 12.0
CL	20.0 ± 2.0	1.6 ± 0.3	67.0 ± 5.4	3.0 ± 0.2

Data are presented as mean values ±standard deviation.

Compared to the pure MCC film, the addition of the CL extract in the MCC:CL biocomposite film resulted in a decrease in the tensile strength but an increase in the elongation at break. When the volume ratio of MCC:CL ranged from 9:1 to 4:6, the tensile strength of the film decreased from 35.3 ± 2.7 MPa to 13.9 ± 2.2 MPa, while the elongation at break increased from 8.60 ± 1.2% to 28.6 ± 2.7%. The reason for this decrease in the tensile strength of the MCC:CL biocomposite films may be attributed to the decrease in the crystallinity of the films (Zhang et al., 2018). The results of the tensile strength of the biocomposite films are correlated with the changes of crystallinity studied before in this article (*The Crystal Structure Analysis of MCC:CL Biocomposite Films*). Atta et al. (2021) also reported a similar tensile strength behavior for similar products, where the addition of 30 wt.% glycerol as the plasticizer in a BC/CMC biocomposite film decreased the tensile strength of the film but increased the elongation at break of the film. This is probably because plasticizers of small molecular weight (such as polysaccharides, pectins, waxes, and so on) can be easily inserted between the cellulose chains, producing a cross-linker effect that weakens the interaction between the cellulose chains, which reduces the mechanical strength and enhances the flexibility of the biocomposite films (Lourdin et al., 1997). It can be concluded that the addition of the CL extract considerably contributed to enhancing the plasticizing behavior of the MCC film.

A comparison of the elongation versus Young's modulus results of the biocomposite film with those of other materials is shown in Figure 6B. The elongation versus Young's modulus results for elastomers, starch-based materials, and engineering polymers materials are listed in Supplementary Table S1. The films in this study have mechanical properties that are comparable to those of common elastomers, starch-based materials, and engineering polymers. The developed films fill the performance gap between synthetic polymers and starch-based polymers, overcoming the performance shortfalls of natural polymers. Thus, the results of this study provide a theoretical basis for increasing the application range of the biocomposite films.

FT-IR spectroscopy was carried out to further investigate the chemical structure of the CL extract, as well as the chemical interaction between the CL extract and MCC in biocomposite films (as shown in Figure 6C). Obviously, the FT-IR spectrum of the CL extract showed the characteristic peaks of cellulose and lignin. The peaks at 3,280, 2,920, 1,670, and 1,020 cm^−1^ were ascribed to the stretching vibration of -OH, the stretching vibration of C-H, the fiber -OH of absorbed water, and the stretching vibration of O-C-O, respectively, which can be used as a reference for the cellulose characteristic peak (Yan et al., 2009; Sun et al., 2013). The peak at 1,530 cm^−1^ corresponded to the aromatic C=C stretching from the aromatic ring of lignin; the peaks in the region between 1,430 and 1,130 cm^−1^ were ascribed to the methoxyl O-CH_3_ and C-OH stretching in lignin (Yan et al., 2009; Nazir et al., 2013; Sun et al., 2013).

FT-IR spectroscopy was also used to determine the inter- and intramolecular hydrogen bonding interactions in the MCC:CL biocomposite films. The hydroxyl stretching region of the MCC:CL biocomposite films is given in Figure 6D. The MCC film showed a broad band at 3,336 cm^−1^ which is due to the presence of O-H vibrations in cellulose (Zhang et al., 2005). The broad band at 3,000–3,500 cm^−1^ in the spectra of the CL extract and MCC:CL biocomposite films is attributed to the stretching vibration of free, self-associated, and inter-associated hydroxyl groups of MCC and CL extract, indicating the hydrogen bonding interaction of MCC with the CL extract in the MCC:CL biocomposite films (Hameed et al., 2013). However, the O-H stretching vibration of the biocomposite film shifted to a higher wavenumber region, indicating the existence of hydrogen bonding between the CL extract and MCC hydroxyl groups in the MCC:CL biocomposite films (Hameed et al., 2013).

### Water Vapor Permeability of MCC:CL Biocomposite Films

**FIGURE 7:**
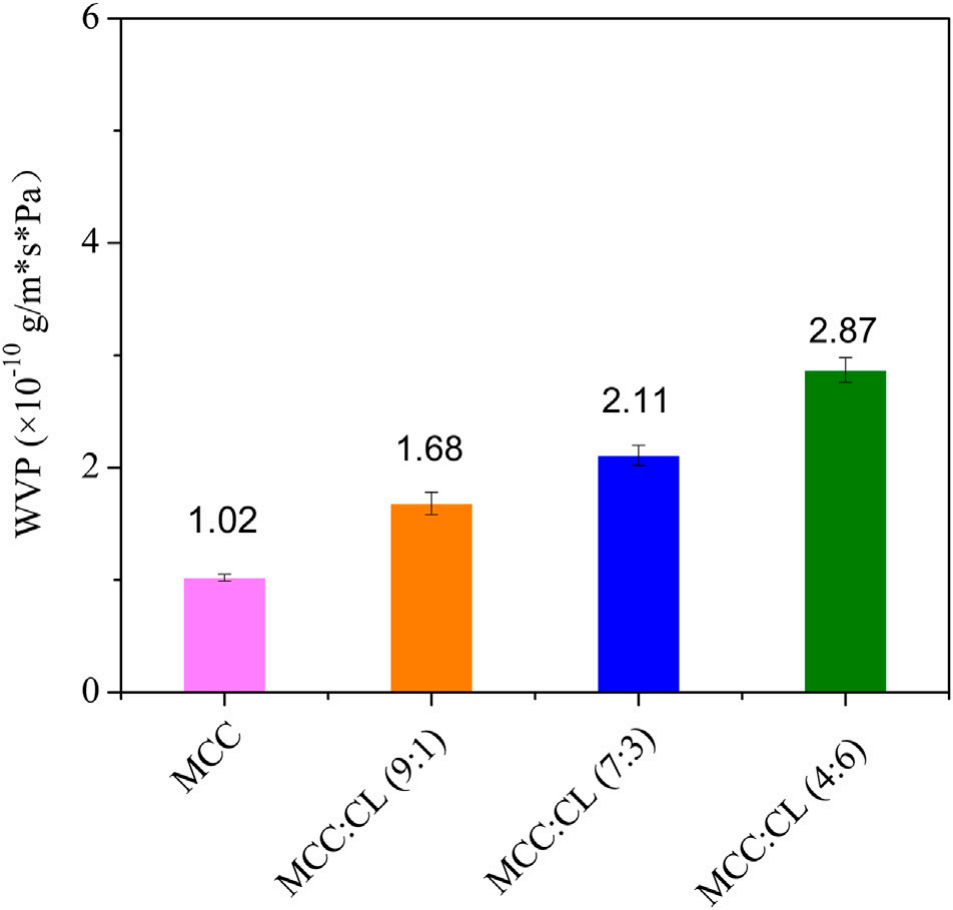
WVP of the MCC film and MCC:CL biocomposite films.

WVP plays an important role in the functioning of biocomposite films. As shown in Figure 7, the WVP value of the pure MCC film was 1.02 × 10^–10^ g/m·s·Pa, while that of the MCC:CL films varied from 1.68 × 10^–10^ to 2.87 × 10^–10^ g/m·s·Pa. Compared with the MCC film, the WVP value of the MCC:CL film (9:1), MCC:CL film (7:3), and MCC:CL film (4:6) was considerably increased by 64.7, 107, and 181%, respectively.

Based on these results, it can be concluded that CL extract causes the MCC film to have a higher WVP. In addition to containing cellulose and lignin, CL extracts may also contain low-molecular-weight substances (polysaccharides, pectins, waxes, and so on); therefore, these extracts can easily penetrate the MCC film structure. Additionally, because it increases the number of hydroxyl groups in the MCC structure, the film becomes very hydrophilic, causing an increase in WVP. Another explanation is that with the addition of CL extract, the network in the MCC film may become less dense, as the CL extract expands the cellulose network, thus increasing the free-volume inter- and intramolecular chains of the MCC film (Martelli et al., 2006). The consequences of the plasticizing action of the CL extract are beneficial to the adsorption and absorption of water molecules on the film; thus, the MCC:CL film exhibits a substantially increased WVP (Irissin-Mangata et al., 2001).

### UV-Shielding Performance of MCC:CL Biocomposite Films

UV light can induce free radical formation that accelerates the oxidation of lipids and destruction of nutrients (Goudarzi et al., 2017). Blocking UV radiation helps prevent the deterioration of products in a package. Transparency is also useful in order to observe changes in the appearance of products in a package. Hence, UV and visible light transmittance and absorption of the MCC film and MCC:CL biocomposite films were investigated.

**FIGURE 8:**
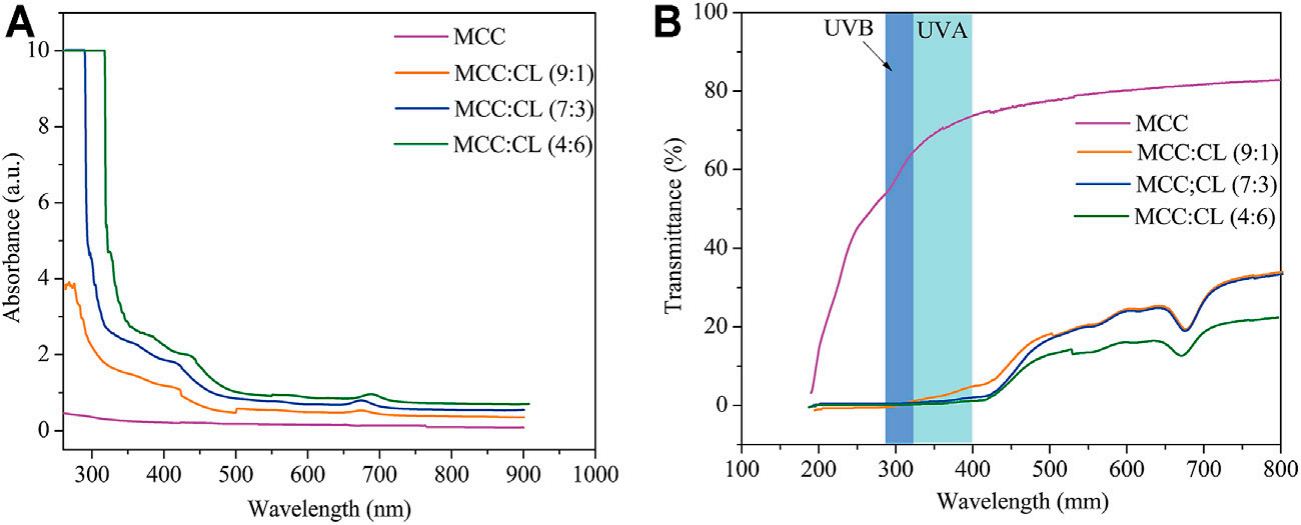
UV and visible light absorbance **(A)** and transmittance **(B)** of the MCC film and MCC:CL biocomposite films.

Figure 8A shows the UV and visible light absorbance spectra of the MCC and MCC:CL biocomposite films. The MCC film showed no UV-shielding ability. By contrast, for the MCC:CL biocomposite films, strong absorption could be observed in the 260–400 nm range, which was associated with the UV-shielding of lignin (Sirviö et al., 2020). This result confirms the good UV-shielding ability of the prepared films, broadening their potential use as functional films.

Figure 8B presents the UV and visible light transmittance spectra of the MCC and MCC:CL biocomposite films. Owing to strong absorption in the 260–400 nm range, the transmittance of UV (<400 nm) was practically zero in the MCC:CL biocomposite films, indicating that the MCC:CL films are excellent UV barriers. Table 2 lists the percentage of UV-A and UV-B radiation blocking and UPF of the MCC and the MCC:CL biocomposite films. The UPF values of the MCC film was very low (approximately 1.63). Approximately 96.4% of UV-B and 98.9% of UV-A light were shielded by the MCC:CL (9:1) film. With increasing CL extract, the UPF of the MCC:CL (7:3) film was as high as 718.8. It is worth noting that the MCC:CL films have a relatively high UV-shielding efficiency and wider UV-shielding bands, suggesting their potential application as UV-shielding films. However, when the CL extract content is increased further, the MCC:CL biocomposite films show a reduction in visible light transmittance (400–760 nm), indicating a poor transparency. This can be attributed to the presence of lignin, which causes the MCC:CL biocomposite films to become brown (Zhang et al., 2019).

**TABLE 2:** Percentage of blocking from UV-A, UV-B, and UPF values of the MCC film and MCC:CL biocomposite films.

Sample	Percentage blocking		UPF value
	UV-A	UV-B	
MCC	30.3	40.7	1.6
MCC:CL (9:1)	96.4	98.9	69.4
MCC:CL (7:3)	99.2	99.9	718.8
MCC:CL (4:6)	99.5	99.9	1,470.0

### Thermogravimetric Analysis of MCC:CL Biocomposite Films

**FIGURE 9:**
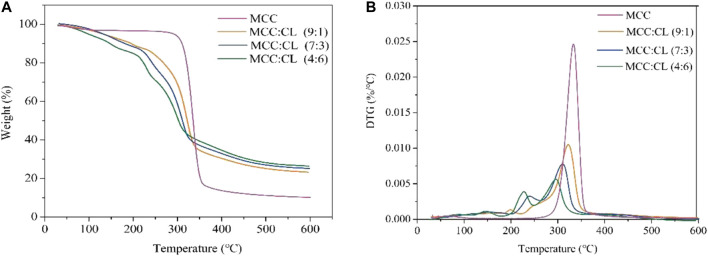
Typical TGA and DTG curves for the MCC film and MCC:CL biocomposite films.

To investigate the thermal properties of the MCC and MCC:CL biocomposite films, a thermogravimetric analysis (TGA/DTG) was performed, as shown in Figure 9. The thermal degradation data of the MCC and MCC:CL biocomposite films are tabulated in Table 3, using the temperatures at which maximum degradation of the samples occurred and the residual weight at 600°C.

**TABLE 3:** Thermal properties of the MCC film and MCC:CL biocomposite films.

Sample	Maximum degradation temperature (°C)		Residual weight % at 600°C
	T_max_		
MCC	335		11.7
MCC:CL (9:1)	322		26.8
MCC:CL (7:3)	311		28.8
MCC:CL (4:6)	300		34.2

From Figure 9A, the initial 6–10% mass loss for the MCC:CL biocomposite films up to about 120–150°C corresponded to the loss of water. Thermal degradation through a one-step process was observed for the MCC film with a decomposition temperature of about 335°C. For the MCC:CL biocomposite films, the onset of degradation of the MCC:CL (9:1), MCC:CL (7:3), and MCC:CL (4:6) films was at about 252, 241, and 233°C, respectively. This result would be related to the degradation of some hemicelluloses and lignin in the CL extract of the MCC:CL biocomposite films (Teaca et al., 2013). The MCC:CL (9:1), MCC:CL (7:3), and MCC:CL (4:6) films showed the maximum degradation temperature at about 322, 311, and 300°C, respectively. The maximum decomposition temperature of the MCC:CL biocomposite films decreased with an increase in the CL extract volume ratio, probably due to the weakening of intermolecular hydrogen bonds between the cellulose and CL extract (Teaca et al., 2013). The results of the TGA suggested that all the MCC:CL biocomposite films exhibited lower thermal stability than the MCC film, implying that the incorporation of the CL extract reduced the thermal stability of the MCC films. The residual weight of the MCC:CL biocomposite films beyond 600°C was higher than that of the MCC film (Table 3). This result is probably because of the presence of lignin from the CL extract, which has an intrinsic flame-resistant property (Mohamad Haafiz et al., 2013).

## Conclusion

In this study, MCC:CL biocomposite films were prepared using CL and MCC *via* direct immersion in TFA. It was possible to increase the flexibility of the MCC films by using CL extract as a plasticizer.

As for the mechanical properties of the MCC:CL biocomposite film, when the volume ratio of MCC:CL ranged from 9:1 to 4:6, the tensile strength of the film decreased from 35.3 ± 2.70 to 13.9 ± 2.2 MPa, while the elongation at break increased from 8.60 ± 1.20 to 28.6 ± 2.70%, indicating that the flexibility of the MCC film improved with increasing CL extract content. The FT-IR spectra proved that the increased flexibility of the MCC:CL biocomposite films may be related to the hydrogen bonding between the CL extract and cellulose. The good compatibility of MCC and CL extract in the MCC:CL biocomposite film was demonstrated using SEM and AFM. The results of X-ray diffraction analysis showed that CL extract introduced in MCC films affected their crystalline structure. Moreover, the reason behind the decrease in tensile strength of the MCC:CL biocomposite films was attributed to the decrease in their crystallinity. Increasing the CL extract volume ratio in MCC:CL biocomposite films enhanced the films’ WVP and UV-shielding efficiency but weakened their thermally stability. When the volume ratio of the MCC:CL was 7:3, the MCC:CL biocomposite film exhibited the best comprehensive performance in terms of WVP (2.11 × 10^–10^ g/m·s·Pa), elongation at break (13.2 ± 1.8%), tensile strength (24.7 ± 2.5 MPa), and UPF values (718.8). To summarize, such MCC:CL biocomposite films have an environmentally friendly nature and can be used for different packing applications.

## Data Availability

The original contributions presented in the study are included in the article/Supplementary Material; further inquiries can be directed to the corresponding authors.

## Footnotes

1
https://www.originlab.com/index.aspx?go=PRODUCTS/Origin
