# Supplementary material for: Preparation and Characterization of Chinese Leek Extract Incorporated Cellulose Composite Films
Source: Front Bioeng Biotechnol. 2021 Nov 15;9:731749. doi: 10.3389/fbioe.2021.731749 (PMC8634590; doi:10.3389/fbioe.2021.731749)
Supplement: Supplementary file 1 [file Table1.DOCX]

**Preparation and Characterization of Chinese Leek Extract Incorporated Cellulose Composite Film**

**Qiying Zeng^1^, Naiyu Xiao^1^,*, Xueqin Zhang^1^,*, Wenhan Luo^1^, Gengshen Xiao^1^，Wanjing Zhai^1^, Le Zhong^1^, Bifeng Lan^2^**

^1^College of Light Industry and Food Science, Zhongkai University of Agriculture and Engineering, Guangzhou 510225, Guangdong, China

^2^Guangzhou Furui High Energy Technology Co., Ltd, Guangzhou 511466, Guangdong, China

*** Correspondence:**

Naiyu Xiao

[xiaony81@163.com](mailto:xiaony81@163.com)

Xueqin Zhang

[zhangxueqin0228@163.com](mailto:zhangxueqin0228@163.com)

**Table S1.** The Elongation versus Young’s modulus for elastomers, starch-based materials and engineering polymers

| Sample | Elongation | Young’s modulus (MPa) |
| --- | --- | --- |
| Elastomers  (Bayer et al., 2014) | 1-10 | 0-100 |
| Starch-based materials  (Bayer et al., 2014) | 0.01-0.05 | 10-500 |
| Engineering polymers  (Bayer et al., 2014) | 0.5-10 | 100-10000 |

**Reference**

Bayer, I.S., Guzman-Puyol, S., Heredia-Guerrero, J.A., Ceseracciu, L., Pignatelli, F., Ruffilli, R., Cingolani, R., and Athanassiou, A. (2014). Direct Transformation of Edible Vegetable Waste into Bioplastics. *Macromolecules* 47**,** 5135-5143. doi: 10.1021/ma5008557
